# A Case of Anti-vascular Endothelial Growth Factor Therapy-Related Nephrotic Syndrome With Marked Intraglomerular Macrophage Infiltration

**DOI:** 10.7759/cureus.50496

**Published:** 2023-12-14

**Authors:** Sho Obara, Tsutomu Inoue, Yusuke Watanabe, Tetsuya Hamaguchi, Tomohiro Ikezawa, Hiroaki Amano, Keisuke Ishizawa, Hirokazu Okada

**Affiliations:** 1 Department of Nephrology, Saitama Medical University, Saitama, JPN; 2 Department of Gastroenterological Oncology, Saitama Medical University International Medical Center, Saitama, JPN; 3 Division of Diagnostic Pathology, Department of Pathology, Saitama Medical University, Saitama, JPN

**Keywords:** glomerular microangiopathy, renal biopsy, bevacizumab toxicity, vascular endothelial growth factor, nephrotic syndrome, foam cells

## Abstract

A 75-year-old woman with colon cancer and distant metastases was treated with fluorouracil, levofolinate, and irinotecan (FOLFIRI) plus bevacizumab postoperatively. During the 32nd course, the patient developed massive proteinuria, and only bevacizumab was discontinued; the proteinuria improved rapidly over time. However, more than six months later, the patient developed massive proteinuria again, and her renal function declined. Renal biopsy revealed glomerular microangiopathy with prominent foam cell infiltration into the glomerulus, which was thought to be caused by chronic endothelial cell damage to the glomerular capillaries. Endothelial cell damage is thought to be caused not only by the inhibition of vascular endothelial growth factor action of bevacizumab in the glomerular capillary but also by the cytotoxicity of the concomitant anticancer drugs and coexisting clinical conditions such as dyslipidemia and hypertension. After discontinuing anticancer agents and intensifying diet and antihypertensive therapy, proteinuria and dyslipidemia slowly improved; however, it became difficult to continue adequate chemotherapy, and the tumor marker levels worsened.

Combination therapies, including molecular targeted agents, have become common, and the side effects of anticancer agents are expected to continue to be complicated. To prevent the onset and severity of renal complications, management of blood pressure, lipid level, and glucose metabolism, as well as multidisciplinary medical management, including dietary therapy, is required.

## Introduction

The protein filtration barrier in the glomerulus comprises the glomerular capillary endothelial cells, glomerular basement membrane, glomerular epithelial cells, and podocytes [1]. Vascular endothelial growth factor (VEGF)-A, which is mainly produced by the glomerular epithelial cells, acts on the VEGF receptors (VEGFR-2) on the surface of nearby glomerular capillary endothelial cells and plays an important role in maintaining normal endothelial cell function through nitric oxide synthesis and maintenance of the cytoskeleton [2]. VEGF also affects angiogenesis, which is closely associated with proliferative diabetic retinopathy and malignant tumor progression [2].

Bevacizumab, which is widely used in the treatment of diabetic retinopathy and malignancy, is a humanized IgG1 antibody that acts via the inhibition of angiogenesis [3]. The most frequent kidney-related adverse effects of bevacizumab include elevated blood pressure and proteinuria [4,5]. Glomerular capillary endothelial cell damage is the predominant pathology, and thrombotic microangiopathy-like changes or glomerular microangiopathies are frequently observed in renal biopsy [6,7]. However, there are cases in which proteinuria may progress to nephrotic syndrome or may be accompanied by focal segmental glomerulosclerosis-like histopathological changes [6] with persistent proteinuria. In recent years, there have been numerous reports on regimens combining bevacizumab with other chemotherapeutic agents. However, combination therapies have been reported to increase the risk of proteinuria [8].

Herein, we report a case of ascending colon cancer with distant metastases that was treated using a multidrug regimen including bevacizumab. During treatment, the patient developed proteinuria, which worsened twice; renal biopsy at the second worsening showed marked foam cell infiltration into the glomerular capillaries, suggesting chronic damage to the glomerular capillary endothelial cells.

## Case presentation

The patient was a 75-year-old woman with the chief complaint of edema on both the lower legs. She had a history of cerebral infarction at the age of 67 years and has been on treatment with acetylsalicylic acid and amlodipine besilate since then. Two years before admission to our department, the patient visited a hospital near her home with the chief complaint of abdominal discomfort and was referred to another cancer hospital for a thorough examination after noticing ascites. At that hospital, a 30 cm ovarian tumor occupying the abdominal cavity and multiple masses, including those in the lung, liver, and colon, were found; the patient was preoperatively diagnosed with stage IV ovarian cancer and underwent bilateral adnexectomy. However, the pathological findings of the resected ovaries revealed metastatic adenocarcinoma of gastrointestinal origin, and together with the preoperative colonoscopy findings, the final diagnosis was ovarian metastasis (and multiple metastases, including peritoneal dissemination) of ascending colon cancer. She developed postoperative ileus and underwent partial jejunal resection with colostomy, and a subcutaneous implantable port was simultaneously created.

**Figure 1:**
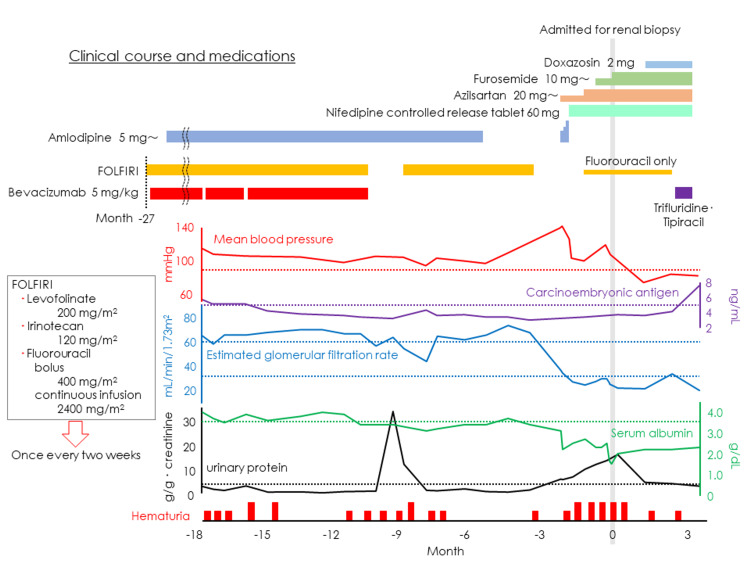
Clinical course and medications Before initiating chemotherapy, proteinuria was 30 mg/dL (qualitative test). During the bevacizumab administration period, the average was 1.308 ± 0.967 g/gCr, peaking at 33.33 g/gCr. However, two months after discontinuing bevacizumab, it decreased to 1.3 g/gCr. As shown in the graph, proteinuria gradually increased starting three months prior to hospitalization, with a second peak at 15.85 g/gCr. Subsequently, it showed a decreasing trend but did not return to baseline levels, persisting between 3.0 and 4.0 g/gCr, indicative of nephrotic-level proteinuria. The black dotted line in the graph represents 3.5 g/gCr.

Thereafter, she received fluorouracil, levofolinate, and irinotecan (FOLFIRI) + bevacizumab therapy at the same hospital, but anti-VEGF therapy (bevacizumab) was discontinued because of the onset of massive proteinuria (after a total of 32 cycles, 10 months before admission in our hospital). After discontinuing bevacizumab, the proteinuria improved rapidly, and the patient was treated with FOLFIRI alone (see Figure 1 on Clinical Course).

Two months prior to admission to our hospital, the patient was admitted to the cancer hospital with sudden leg edema and elevated blood pressure (mean systolic blood pressure: 150 → 200 mmHg). She experienced a recurrence of massive proteinuria upon admission, accompanied by acute kidney injury. A nephrologist at the same hospital treated her concurrently and started antihypertensive therapy; however, she had worsening hypoalbuminemia and proteinuria. The patient was referred to our department for a thorough examination, including a renal biopsy.

On admission, the patient’s physical examination findings were as follows: height, 153.0 cm; weight, 60.4 kg; body mass index, 25.8; body temperature, 36.4 °C; blood pressure, 153/87 mmHg; pulse, 72 beats/min; respiratory rate, 16 breaths/min, and no abnormal findings in the head and neck. Alveolar breath sounds were normal without rales, and the heart sounds (I and II) were normal without murmurs. Upon examination, there were no subjective symptoms, the abdomen was flat and soft, bowel peristalsis was normal, and no abnormal findings were noted. Moderate edema was noted in both the lower legs; however, there was no obvious difference between the right and left legs. Chest radiography revealed a cardiothoracic ratio of 56.2% and no pleural effusion or infiltrative shadows in the lung fields. A 12-lead electrocardiogram showed normal sinus rhythm, a heart rate of 92 beats/min, and no ST-T changes. A non-contrast-enhanced X-ray computed tomography of the abdomen showed mild atrophy of the bilateral kidneys. No dilatation of the renal pelvis or ureter was observed, and we considered that these changes corresponded to chronic kidney disease. The laboratory findings on admission are presented in Table 1.

**Table 1:** Results of urine and blood tests at admission for renal biopsy

Test	Results	Units	Reference value
Urinalysis			
Specific gravity	1.016		1.010-1.025
pH	5.5		4.5-7.5
Sugar	100	mg/dL	<30
Ketone body	Negative		Negative
Urobilinogen	Normal		Normal
Bilirubin	Negative		Negative
Occult blood	1+		Negative
Urine protein/creatinine ratio	11.85		<0.15
Urinary sediment			
Red blood cell	1-4	/HPF	<4
White blood cell	20-29	/HPF	<4
Oval fat body	50-99	/HPF	<1
Granular cast	10-19	/HPF	<1
Fatty cast	10-19	/WF	<1
Urinary N-acetyl-β-D-glucosaminidase	58.2	IU/L	<11.2
Urinary β2-microglobulin	7285	μg/L	<230
Complete blood cell count			
White blood cell	4010	/μL	3500-9100
Neutrophil	60.9	%	40.0-74.0
Lymphocyte	30.7	%	18.0-59.0
Monocyte	5.2	%	0.0-8.0
Eosinophil	3.2	%	0.0-6.0
Basophil	0	%	0.0-2.0
Red blood cell	3.11	x10^6^/μL	3.76-5.00
Hemoglobin	9.1	g/dL	11.3-15.2
Hematocrit	27.8	%	33.4-44.9
Mean corpuscular volume	89.4	fL	79.0-100.0
Mean cell hemoglobin	29.3	pg	26.3-34.3
Mean cell hemoglobin concentration	32.7	%	30.7-36.6
Platelet	20.2	x10^4^/μL	13.0-36.9
Serum chemistry			
Total protein	4.8	g/dL	6.7-8.3
Albumin	2.3	g/dL	3.8-5.2
Aspartate aminotransferase	16	U/L	10-40
Alanine aminotransferase	10	U/L	5-40
Lactate dehydrogenase	262	U/L	124-222
Alkaline phosphatase	303	U/L	38-113
Creatinine	1.54	mg/dL	0.47-0.79
Estimated glomerular filtration rate	25.9	mL/min/1.73 m^2^	>60
Uric acid	6.6	mg/dL	2.5-7.0
Blood urea nitrogen	33.1	mg/dL	8.0-22.0
Triglyceride	351	mg/dL	50-149
Total cholesterol	418	mg/dL	150-219
High-density lipoprotein cholesterol	77	mg/dL	40-96
Low-density lipoprotein cholesterol	271	mg/dL	70-139
Sodium	141	mEq/L	136-147
Chloride	111	mEq/L	98-109
Potassium	4.8	mEq/L	3.6-5.0
Adjusted calcium	10.1	mg/dL	8.5-10.2
Inorganic phosphorus	3.8	mg/dL	2.4-4.3
Total bilirubin	0.3	mg/dL	0.3-1.2
C-reactive protein	0.13	mg/dL	<0.14
Iron	32	μg/dL	48-154
Total iron binding capacity	199	μg/dL	246-410
Unsaturated iron binding capacity	167	μg/dL	108-325
Ferritin	257	ng/mL	3.6-114
Immunoglobulin G	421	mg/dL	870-1700
Immunoglobulin A	95	mg/dL	110-410
Immunoglobulin M	60	mg/dL	46-260
Serum complement level	70	U/mL	25-48
Coagulating system			
Fibrinogen	599	mg/dL	150-400
D-dimer	12.26	μg/mL	<1.0
Bleeding time	150	S	<300
Activated partial thromboplastin time	26.7	S	24.3-36.0
Prothrombin time	10.6	S	10.5-13.5
Prothrombin time-international normalized ratio	0.89		0.85-1.15
Endocrine and metabolism			
Hemoglobin A1c	5.7	%NGSP	4.6-6.2
Free triiodothyronine	1.98	pg/mL	2.52-4.06
Thyroid-stimulating hormone	2.31	μIU/mL	0.61-4.23
Free thyroxine	1.08	ng/dL	0.75-1.45
Brain natriuretic peptide	110.4	pg/mL	<18.4
Autoantibody			
Antinuclear antibody	40		<40
Proteinase3-anti-neutrophil cytoplasmic antibody	Negative		Negative
Myeroperoxidase-anti-neutrophil cytoplasmic antibody	Negative		Negative
Infection			
Serological test for syphilis	Negative		Negative
Treponema pallidum	Negative		Negative
Hepatitis B surface antigen	Negative		Negative
Hepatitis C virus antibody	Negative		Negative

The patient had massive proteinuria, hypoalbuminemia, and dyslipidemia, and was diagnosed with nephrotic syndrome. The estimated glomerular filtration rate was less than 30 mL/min/1.73 m^2^, corresponding to chronic kidney disease stage 4A3. All items tested were negative for infections and autoantibodies. A renal biopsy was performed as scheduled.

**Figure 2:**
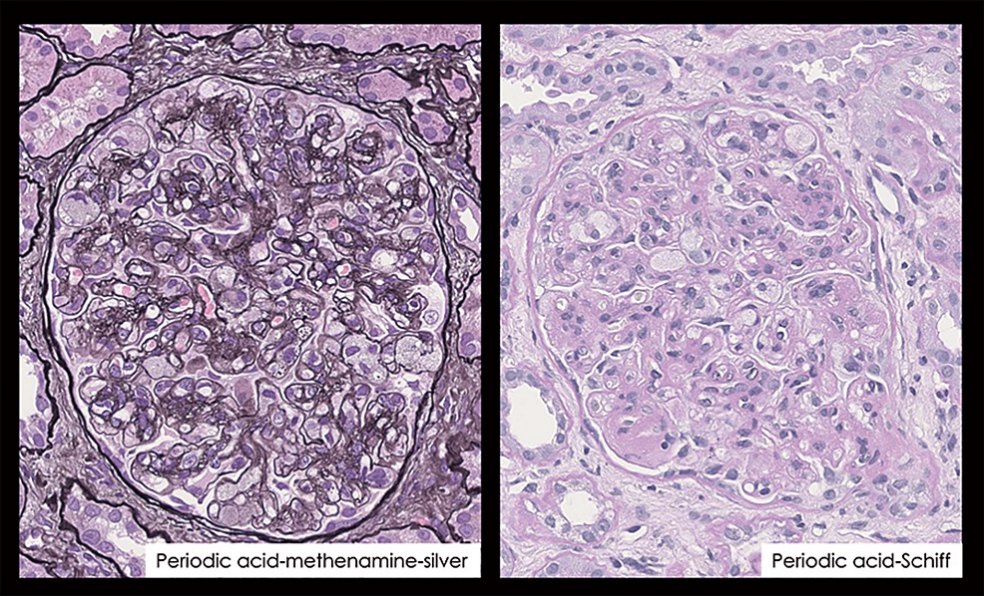
Histopathological findings of renal biopsy Marked infiltration of foam cells is observed. The vessel lumen is narrowed. Objective lens ×40.

The renal biopsy revealed histopathological findings consistent with chronic kidney disease, tubular atrophy, and fibrosis of the interstitium. Three to five glomeruli were observed in each specimen. Periodic acid-methenamine-silver and periodic acid-Schiff staining showed foam cell infiltration into the glomerular capillaries, an enlarged mesangial area, and an enlarged subendothelial lumen with a narrowed glomerular capillary lumen (Figure 2).

**Figure 3:**
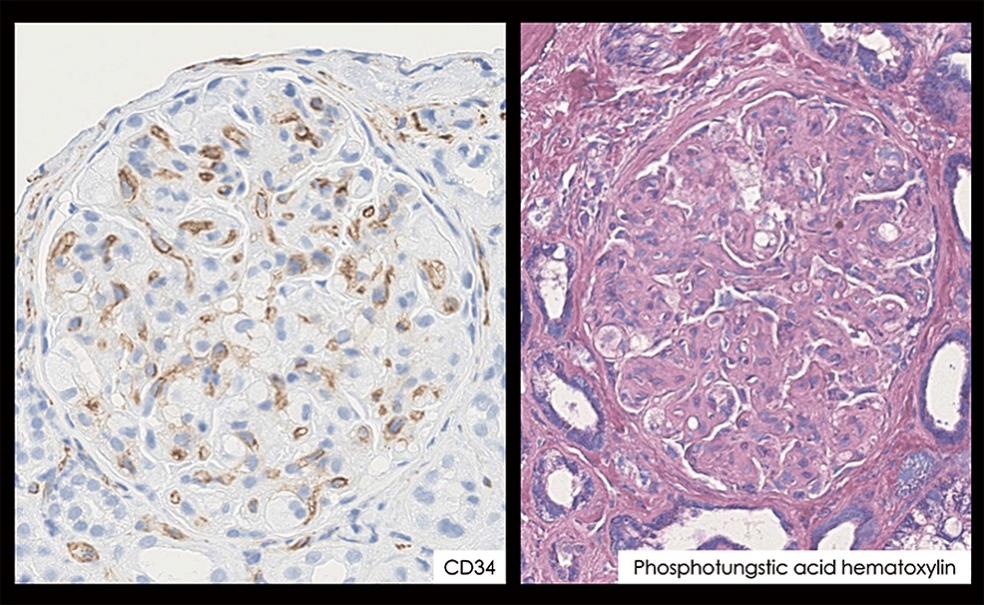
Histopathological findings of renal biopsy In endothelial cell staining, the gap between the endothelial cells and the basement membrane is clearly visible. No obvious thrombi were observed in the glomeruli. Objective lens ×40.

CD34 staining confirmed the presence of a vascular endothelium with similar findings (Figure 3).

**Figure 4:**
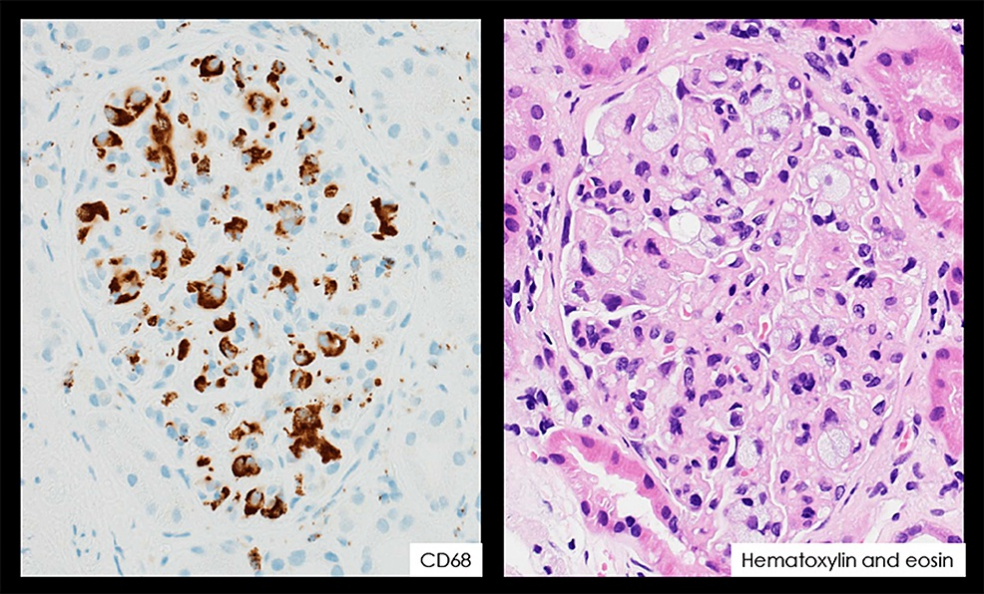
Histopathological findings of renal biopsy The foam cells were CD68-positive macrophages. Objective lens ×40.

The glomerular basement membrane was double-contoured in some areas. In contrast, no spikes were observed. No extracapillary proliferation (i.e., crescentic formation) was observed. Phosphotungstic acid-hematoxylin staining did not reveal any obvious fibrin thrombi in the glomeruli. The foam cells in the glomerular capillaries were CD68-positive and thought to be macrophages (Figure 4).

**Figure 5:**
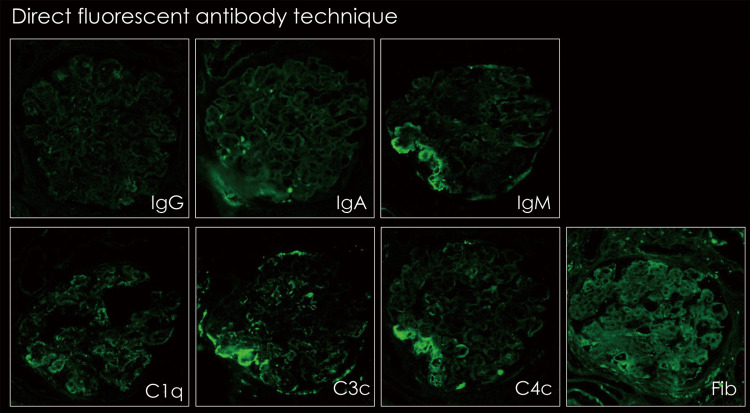
Findings of direct fluorescent antibody staining of renal biopsy IgM and C3 were positive in part of the subendothelium. Objective lens ×40.

Direct fluorescent antibody staining was positive for immunoglobulins in a portion of the glomerular capillary wall; however, no significant complement deposition was observed (Figure 5).

**Figure 6:**
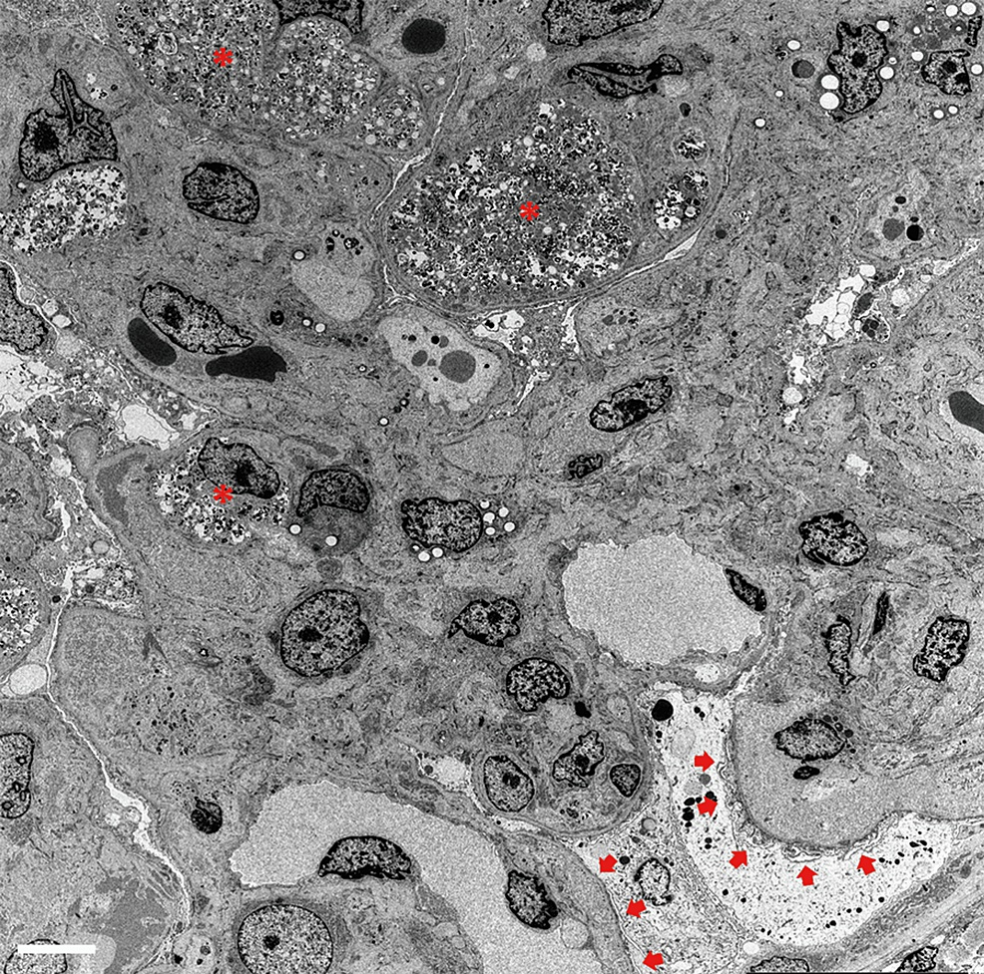
Findings of the renal biopsy under the electron microscope The asterisks indicate foam cells. Arrowheads indicate loss of normal foot processes. Scale bar = 5 μm.

Electron microscopy revealed only a small number of high-electron-density deposits, a finding that is unlikely to be the cause of glomerular changes (Figure 6).

In conclusion, this was not glomerulonephritis due to an immunoglobulin or complement mediation (primary membranoproliferative glomerulonephritis or C3 nephropathy), but rather nephropathy due to glomerular capillary endothelial cell damage than thrombotic microangiopathy. Therefore, the use of bevacizumab was suspected to be the cause.

In addition to increasing the dose of antihypertensive medication before renal biopsy, a low-sodium diet and intensification of diuretics caused a gradual decrease in blood pressure. Simultaneously, dyslipidemia and urinary protein levels improved. Serum albumin levels also showed gradual improvement, and renal function stopped deteriorating. Tumor marker levels slowly worsened, although fluorouracil alone was initiated before the renal biopsy. The patient was switched to a fixed-dose combination of trifluridine and tipiracil; however, this was unsuccessful. The treatment plan was reviewed as necessary with a view to switching to the best supportive care.

## Discussion

In this case of nephrotic syndrome with prominent macrophage infiltration of the glomerular capillaries, we considered glomerular microangiopathy related to the anti-VEGF therapy (bevacizumab) as a possible cause.

The patient had two exacerbations of proteinuria; the first exacerbation occurred during combination (FOLFIRI+bevacizumab) therapy, approximately 10 months before admission for renal biopsy. The patient had severe proteinuria without a decrease in serum albumin level, suggesting that massive proteinuria occurred acutely. The rapid improvement after discontinuation of bevacizumab suggested that bevacizumab inhibited VEGF action in the glomerular capillaries. Thereafter, FOLFIRI therapy alone was resumed without bevacizumab; however, approximately six months later, the patient experienced another (second) slow worsening of proteinuria, accompanied by a decline in renal function. At that time, the proteinuria did not improve despite discontinuation of FOLFIRI and intervention with antihypertensive therapy, and a renal biopsy was performed to investigate the cause. The results revealed glomerular damage, characterized by marked macrophage infiltration.

In this case, the narrowing of the glomerular capillary lumen and enlargement of the subendothelial lumen were remarkable, suggesting that glomerular endothelial cell damage was the primary cause of the lesion. Thrombotic microangiopathy is another well-known glomerular vascular lesion, in which glomerular endothelial cell damage is the predominant lesion [9]. Although both glomerular and thrombotic microangiopathies can be drug-induced lesions, their histologies differ. Glomerular microangiopathy is characterized by macrophage infiltration, whereas fibrin thrombi, crushed red blood cells, and onion skin changes, which are observed in thrombotic microangiopathy, are less frequent [9]. The patient was diagnosed with glomerular microangiopathy based on histopathological features.

Foam cells were occasionally observed in renal biopsy specimens. It has been reported that foam cells are observed in clusters in the interstitium of various chronic glomerular lesions, not limited to Alport syndrome [10]. Conversely, foam cells have been observed in the glomerular capillaries, known as “intra-capillary foam cell infiltration,” in focal segmental glomerulosclerosis [11], even if it is not as prominent as in the present case. Detailed studies on the putative mechanism of foam cell formation reported that the foam cell infiltration originates from glomerular epithelial cell damage [12,13]. Since anti-VEGF therapy was introduced into clinical practice, it has become well known that prolonged endothelial cell damage due to the inhibition of VEGF action in the glomerular capillaries is accompanied by irreversible epithelial cell (podocyte) damage and histopathological changes such as focal segmental glomerulosclerosis-like changes, resulting in refractory massive proteinuria [6].

Based on the above findings, we hypothesized that the secondary proteinuria in this case resulted from prolonged endothelial cell damage induced by anti-VEGF therapy (bevacizumab in this case), which led to epithelial cell damage and glomerular microangiopathy with marked macrophage infiltration. Lipid metabolism abnormalities, such as the high low-density lipoprotein cholesterol levels observed in this case, are thought to be involved in macrophage infiltration and foam cell formation. In other words, we thought that the continued use of anti-VEGF agents and other anticancer drugs was responsible for the significant intracapillary foam cell appearance due to endothelial cell/podocyte damage and the production of reactive oxygen species, which caused lipid deposition and oxidation in the glomerular capillaries [13].

It is also interesting to note that in this case, proteinuria worsened again approximately six months after the discontinuation of anti-VEGF therapy. The subsequent disappearance of proteinuria after the first episode of massive proteinuria does not necessarily guarantee the complete recovery of glomerular endothelial cell function. It is possible that the glomerular endothelial cell damage was prolonged or recovery of damage was delayed, even if the amount of proteinuria in the urine collected in the “urinalysis cup” decreased. Although glomerular epithelial cell damage is induced immediately after anti-VEGF antibody administration, there is a time lag of several days before proteinuria increases [14], and the absence of proteinuria does not necessarily reflect the absence of glomerular damage. In addition, small amounts of abnormal proteinuria may be compensated by the reabsorptive actions of normal tubules [1].

The reason for this is unclear, but the possibility that the resumed antineoplastic agents other than anti-VEGF agents may have acted in a cytotoxic manner cannot be ruled out. It has been reported that cancer patients, including those with pre-renal conditions, are prone to developing acute kidney injury [15]. Regarding proteinuria, it is suggested that, in addition to drug-related endothelial cell damage as seen in this case, various secondary glomerular diseases associated with malignancies may also be contributing factors [16]. Recently, a report was published on the progression of renal dysfunction during treatment with the standard triplet regimen, fluorouracil, folinic acid, and oxaliplatin (FOLFOX)/FOLFIRI, with bevacizumab or cetuximab in patients with metastatic rectal colon cancer [17]. According to this report, the decline in renal function during treatment was more pronounced with bevacizumab than with cetuximab, and the combination of FOLFIRI plus bevacizumab resulted in the worst prognosis for renal function, with an incidence of acute kidney injury of 19.2% [17]. It has been suggested that not only anti-VEGF agents but also the type of anticancer drugs used in combination with anti-VEGF agents may be related to the course of renal function. In recent years, many protocols have been proposed to combine multiple antitumor drugs and renal failure is expected to become increasingly complicated.

## Conclusions

We reported a case of glomerular microangiopathy caused by bevacizumab treatment. The clinical presentation was nephrotic syndrome, and a renal biopsy showed prominent macrophage infiltration (foam cells) into the glomerular capillaries. The histopathological findings suggested a chronic course of the disease, and concomitant use of anticancer drugs, dyslipidemia, and elevated blood pressure, in addition to bevacizumab, may have contributed to the prolonged glomerular damage. The use of a combination of anticancer agents may also complicate renal effects compared with the use of a single agent. Careful intervention is required to manage blood pressure and lipid metabolism during long-term cancer chemotherapy.

## References

[1] D'Amico G, Bazzi C (2003). Pathophysiology of proteinuria. Kidney Int.

[2] Estrada CC, Maldonado A, Mallipattu SK (2019). Therapeutic inhibition of VEGF signaling and associated nephrotoxicities. J Am Soc Nephrol.

[3] Garcia J, Hurwitz HI, Sandler AB, Miles D, Coleman RL, Deurloo R, Chinot OL (2020). Bevacizumab (Avastin®) in cancer treatment: A review of 15 years of clinical experience and future outlook. Cancer Treat Rev.

[4] Yang JC, Haworth L, Sherry RM (2003). A randomized trial of bevacizumab, an anti-vascular endothelial growth factor antibody, for metastatic renal cancer. N Engl J Med.

[5] Zhu X, Wu S, Dahut WL, Parikh CR (2007). Risks of proteinuria and hypertension with bevacizumab, an antibody against vascular endothelial growth factor: Systematic review and meta-analysis. Am J Kidney Dis.

[6] Person F, Rinschen MM, Brix SR (2019). Bevacizumab-associated glomerular microangiopathy. Mod Pathol.

[7] Izzedine H, Massard C, Spano JP, Goldwasser F, Khayat D, Soria JC (2010). VEGF signalling inhibition-induced proteinuria: Mechanisms, significance and management. Eur J Cancer.

[8] Wu S, Kim C, Baer L, Zhu X (2010). Bevacizumab increases risk for severe proteinuria in cancer patients. J Am Soc Nephrol.

[9] Pfister F, Amann K, Daniel C, Klewer M, Büttner A, Büttner-Herold M (2018). Characteristic morphological changes in anti-VEGF therapy-induced glomerular microangiopathy. Histopathology.

[10] Wu Y, Chen Y, Chen D, Zeng C, Li L, Liu Z (2009). Presence of foam cells in kidney interstitium is associated with progression of renal injury in patients with glomerular diseases. Nephron Clin Pract.

[11] Nagata M, Kobayashi N, Hara S (2017). Focal segmental glomerulosclerosis; why does it occur segmentally?. Pflugers Arch.

[12] Hara S, Kobayashi N, Sakamoto K (2015). Podocyte injury-driven lipid peroxidation accelerates the infiltration of glomerular foam cells in focal segmental glomerulosclerosis. Am J Pathol.

[13] Kobayashi N, Ueno T, Ohashi K (2015). Podocyte injury-driven intracapillary plasminogen activator inhibitor type 1 accelerates podocyte loss via uPAR-mediated β1-integrin endocytosis. Am J Physiol Renal Physiol.

[14] Sugimoto H, Hamano Y, Charytan D, Cosgrove D, Kieran M, Sudhakar A, Kalluri R (2003). Neutralization of circulating vascular endothelial growth factor (VEGF) by anti-VEGF antibodies and soluble VEGF receptor 1 (sFlt-1) induces proteinuria. J Biol Chem.

[15] Christiansen CF, Johansen MB, Langeberg WJ, Fryzek JP, Sørensen HT (2011). Incidence of acute kidney injury in cancer patients: A Danish population-based cohort study. Eur J Intern Med.

[16] Jhaveri KD, Shah HH, Calderon K, Campenot ES, Radhakrishnan J (2013). Glomerular diseases seen with cancer and chemotherapy: A narrative review. Kidney Int.

[17] Lim AR, Kim JH, Hyun MH, Kim YH, Lee S (2022). Prognostic factors for renal function deterioration during palliative first-line chemotherapy for metastatic colorectal cancer: A retrospective study. Support Care Cancer.

